# Early initiation of breastfeeding and perinatal conditions among mothers in southern Ecuador: a descriptive cross-sectional study

**DOI:** 10.3389/fnut.2026.1876285

**Published:** 2026-09-23

**Authors:** Ana Lizette Rojas-Rodriguez, Danilo Fabricio Agila Sánchez, Víctor Merchán Cuenca, Cristina Elisabeth Urgilés-Barahona

**Affiliations:** Department of Health Sciences, Faculty of Health Sciences, Universidad Técnica Particular de Loja, Loja, Ecuador

**Keywords:** breastfeeding practices, early initiation of breastfeeding, early-life nutrition, first hour of life, maternal and child health, neonatal, perinatal conditions

## Abstract

**Background:**

Early initiation of breastfeeding within the first hour of life is a key indicator of newborn care quality and is recommended because of its relevance for neonatal health, survival, and the successful establishment of breastfeeding. However, its implementation varies across healthcare settings, and local evidence remains important for understanding the perinatal circumstances in which breastfeeding initiation occurs.

**Objective:**

To describe breastfeeding practices during the first hour of life and selected perinatal conditions reported by adult mothers (≥18 years) recruited from four public primary healthcare centers in southern Ecuador.

**Methods:**

A descriptive cross-sectional study was conducted between February and October 2025. Adult mothers (≥18 years) who had given birth within the previous two years were recruited while attending pediatric or gynecological consultations. Data were collected using a structured self-administered questionnaire assessing sociodemographic characteristics, obstetric history, perinatal conditions, and breastfeeding practices during the first hour after birth. Descriptive statistics, including frequencies, percentages, and 95% confidence intervals, were calculated.

**Results:**

A total of 343 mothers participated. Early initiation of breastfeeding was reported by 56.0% of participants, whereas 44.0% reported initiation after the first hour of life. Immediate skin-to-skin contact was reported by 77.8% of mothers, and 33.5% experienced difficulties initiating breastfeeding. Cesarean delivery accounted for 48.1% of births, maternal complications were reported by 21.0% of participants, and neonatal complications by 12.2%. Descriptive stratification showed notable differences in breastfeeding initiation across perinatal conditions. Early initiation was reported by 82.0% of mothers after vaginal delivery compared with 28.0% after cesarean delivery. Similarly, 67.0% of mothers reporting immediate skin-to-skin contact initiated breastfeeding within the first hour, compared with 11.8% of those whose contact occurred after 1 h.

**Conclusion:**

More than four out of every ten mothers in this cohort reported delayed breastfeeding initiation despite the high frequency of immediate skin-to-skin contact. Breastfeeding initiation occurred within a perinatal context characterized by substantial cesarean delivery rates, maternal-reported breastfeeding difficulties, and the presence of maternal and neonatal complications. These findings provide contemporary descriptive evidence on breastfeeding practices in southern Ecuador and support continued monitoring of early breastfeeding initiation as an indicator of maternal and newborn care quality.

## Graphical Abstract

**Figure:**
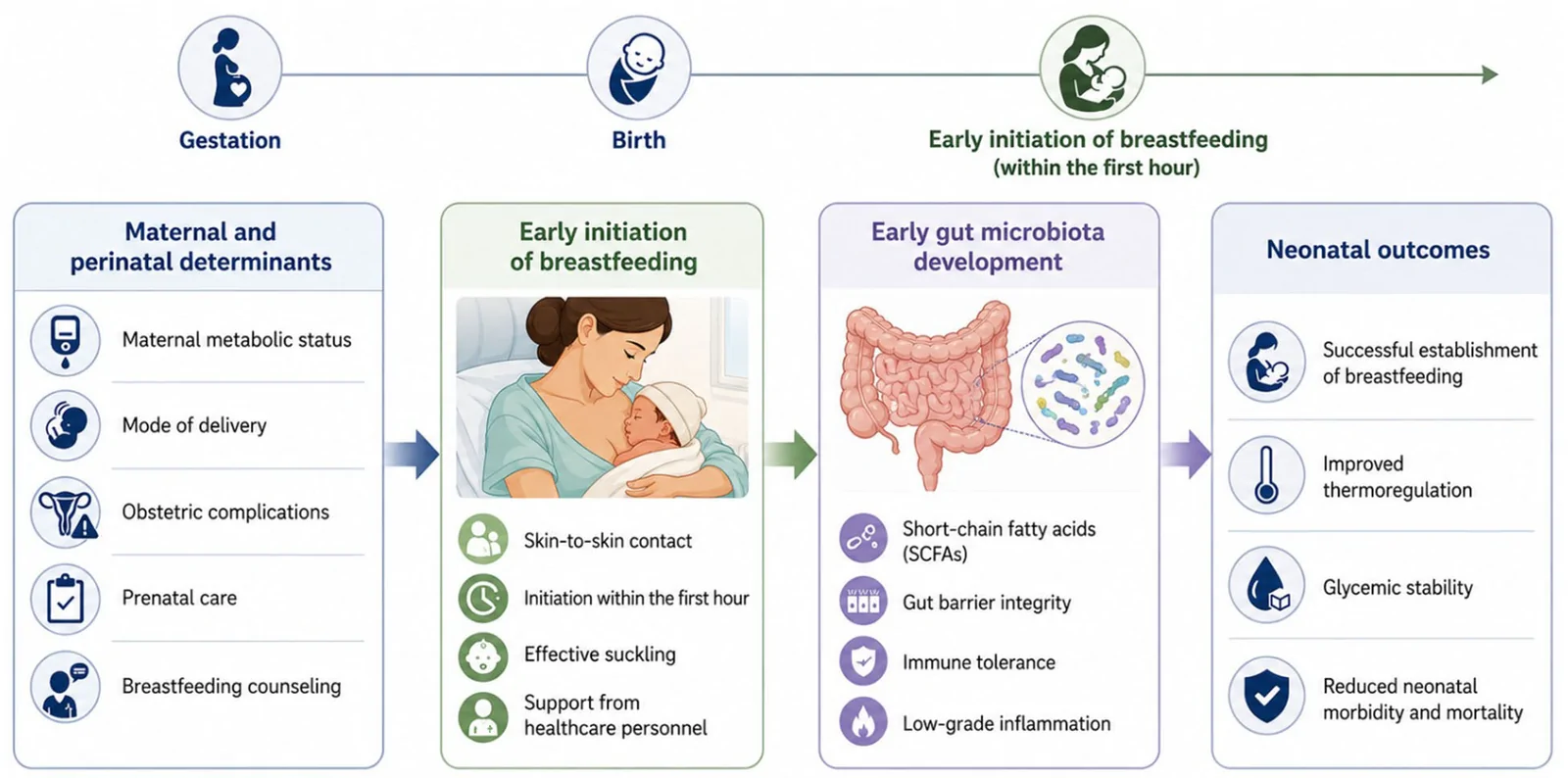

## 1 Introduction

The first hour of life, often referred to as the “golden hour,” represents a critical period in the newborn's transition from intrauterine to extrauterine life. During this period, early initiation of breastfeeding (EIBF) is recommended by the World Health Organization (WHO) and UNICEF because of its well-established benefits for neonatal survival, child health, and the successful establishment of breastfeeding (1–3). EIBF enables newborns to receive colostrum, the first milk, which provides essential nutrients and immunological protection during the early neonatal period. Consequently, breastfeeding within the first hour after birth is considered a key component of essential newborn care and an important indicator of the quality of maternal and neonatal healthcare (1–3).

Beyond its nutritional role, colostrum provides a highly bioactive milieu that supports neonatal adaptation through interconnected cellular and molecular mechanisms. It contains viable immune and stem/progenitor cells, extracellular vesicles, regulatory microRNAs, cytokines, and other bioactive factors involved in intercellular signaling, gene-expression regulation, inflammatory modulation, and immune tolerance (4). At the gastrointestinal level, epidermal growth factor (EGF) promotes epithelial proliferation and differentiation and supports mucosal maturation and barrier integrity, while lactadherin (MFG-E8) modulates intestinal inflammatory signaling through the TLR4/NF-κB pathway. Secretory IgA (sIgA), together with macrophages, neutrophils, lymphocytes, and pro- and anti-inflammatory cytokines, further contributes to mucosal defense and neonatal immune regulation. In parallel, growth and neurotrophic factors and bioactive lipids contribute to intestinal maturation and support vascular and nervous system development (5). Collectively, these cellular and molecular mechanisms provide biological plausibility for the benefits of early colostrum exposure during the critical transition to extrauterine life.

Despite the well-established benefits of early colostrum exposure and international recommendations supporting breastfeeding initiation within the first hour after birth, the prevalence of EIBF remains suboptimal in many regions of the world. Recent estimates indicate that fewer than half of newborns initiate breastfeeding within the first hour of life, with reported prevalences ranging widely across countries and healthcare settings (3, 6, 7). In low- and middle-income countries, many nations continue to report EIBF rates below recommended targets (7, 8).

In Latin America, breastfeeding is widely recognized as a beneficial and culturally accepted practice; however, timely initiation remains variable across settings (8–11). In Ecuador, the National Health and Nutrition Survey (ENSANUT) reported that 72.7% of children were breastfed within the first hour of life. Although national estimates are available, local descriptive evidence characterizing breastfeeding practices and the perinatal context in which breastfeeding is initiated remains limited in Ecuador (12).

Early initiation of breastfeeding is influenced by multiple maternal, socioeconomic, 5cultural, institutional, and healthcare-related factors. Previous studies have described variability in early breastfeeding initiation according to maternal, neonatal, socioeconomic, institutional, and healthcare-related characteristics. These studies have reported differences in breastfeeding initiation across perinatal circumstances such as mode of delivery, maternal and neonatal complications, immediate mother–infant contact after birth, and healthcare practices implemented during the immediate postnatal period (8–11). Cultural beliefs regarding newborn feeding and institutional practices, such as routine mother–infant separation, have also been described in relation to breastfeeding initiation (13–16).

Therefore, describing breastfeeding practices during the first hour of life and the perinatal circumstances in which breastfeeding is initiated remains important from both clinical and public health perspectives. The aim of this study was to describe breastfeeding practices during the first hour of life and selected perinatal conditions among mothers recruited through four public primary healthcare centers in an urban area of southern Ecuador.

## 2 Materials and methods

### 2.1 Study type and research design

This manuscript is part of a larger research project developed between February and October 2025. The present article reports the findings of its descriptive phase, which aimed to characterize breastfeeding practices during the first hour of life among mothers attending four primary healthcare centers in the city of Loja, southern Ecuador.

### 2.2 Participant recruitment

The study was conducted in four primary healthcare centers belonging to the Ecuadorian Ministry of Public Health and located in the city of Loja, southern Ecuador. These centers were intentionally selected because they provide healthcare services to one of the largest maternal and child populations in the region and were therefore considered appropriate settings for recruiting the study population. They offer comprehensive maternal and child healthcare services, including antenatal care, postnatal care, gynecological consultations, and pediatric follow-up.

The study population comprised 533 mothers registered in the four participating primary healthcare centers. Eligibility was assessed according to predefined inclusion and exclusion criteria. Eligible participants were mothers aged 18 years or older attending pediatric or gynecological consultations who had given birth within the previous 2 years. This time frame was selected because the World Health Organization recommends continued breastfeeding up to 2 years of age or beyond; therefore, mothers of children younger than 2 years constitute a particularly relevant population for the study of breastfeeding-related practices. Women younger than 18 years of age were excluded because of ethical considerations and the potential influence of age-specific psychosocial factors on breastfeeding practices.

The study aimed to recruit all eligible mothers identified during the recruitment period. Recruitment was conducted between February and October 2025. Among the 533 mothers registered in the participating healthcare centers, 51 did not attend their scheduled appointments, 44 had children older than 2 years at the time of the survey, 57 were younger than 18 years of age, and 38 declined to participate. The final analytical sample consisted of 343 mothers (Figure 1).

**Figure 1:**
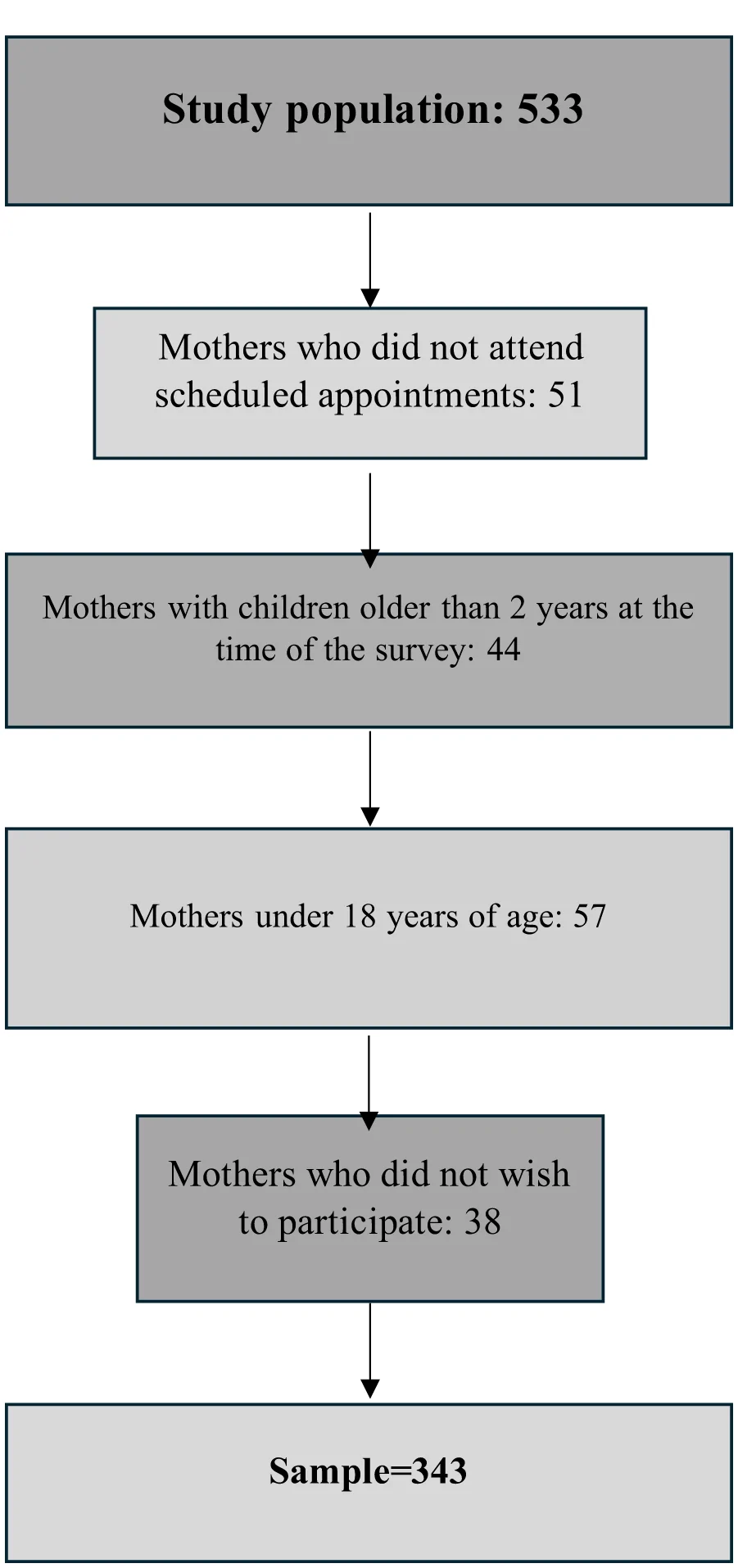
Flow diagram of participant recruitment and final sample selection for the study on early initiation of breastfeeding.

Although the study aimed to recruit all eligible mothers, a minimum sample size of 222 participants was calculated using the formula for estimating a proportion in a finite population (95% confidence level; 5% margin of error). The final analytical sample (*n* = 343) exceeded the calculated minimum sample size.

The study was conducted in accordance with the ethical principles for research involving human participants and received approval from the Research Ethics Committee of the Universidad Técnica Particular de Loja (CEISH-UTPL) (approval code: 2025-01-INT-EO-RM-005). All procedures complied with the ethical standards established in the Declaration of Helsinki.

Prior to participation, all eligible mothers received detailed information about the study and provided written informed consent. Participants were informed that they could decline participation or withdraw from the study at any time without any consequences for their medical care or access to health services. No financial compensation, incentives, or other material benefits were provided for participation.

### 2.3 Instruments

Data were collected using a structured, self-administered questionnaire developed *ad hoc* by the research team. The instrument was designed based on a review of the scientific literature on early initiation of breastfeeding and current international recommendations to identify relevant breastfeeding practices and perinatal conditions during the first hour after birth.

The first section included sociodemographic variables and was developed in accordance with the structure of national survey instruments used by the Ecuadorian National Institute of Statistics and Census (INEC). The second section collected information on obstetric history, maternal and neonatal clinical conditions, delivery-related factors, and breastfeeding practices during the immediate postpartum period.

The final instrument consisted of 33 multiple-choice items organized into four conceptual domains: (1) sociodemographic characteristics, (2) obstetric history, (3) medical and delivery-related factors, and (4) breastfeeding practices and experiences during the first hour after birth. All variables were categorical, allowing standardized coding and descriptive analysis. The questionnaire was specifically designed to characterize breastfeeding practices and perinatal conditions related to the first hour after birth in the study population.

Breastfeeding initiation timing was assessed through maternal self-report. Mothers were asked: “How long after birth did you first breastfeed your baby?” Response options included predefined time categories (within the first 5 min, within the first 10 min, within the first hour, and after the first hour). For descriptive analyses, responses were categorized as early initiation of breastfeeding (within the first hour after birth) and delayed initiation of breastfeeding (after the first hour).

The content validity of the questionnaire was assessed by a panel of five experts in pediatrics, neonatology, lactation, epidemiology, and maternal-child health. Each expert independently assessed the relevance, clarity, and appropriateness of the items using a structured evaluation matrix with an ordinal scale. Based on their feedback, minor revisions were made to improve the conceptual clarity and coherence of the instrument. Content validity was quantified using Lawshe's method, calculating the Content Validity Ratio (CVR) for each item and the overall Content Validity Index (CVI). The instrument achieved a CVI of 0.83, indicating adequate content validity for health research instruments.

In addition, a pilot comprehension assessment was conducted with 50 mothers with characteristics like those of the target population attending primary healthcare services. The assessment was performed to evaluate the clarity and comprehensibility of the questionnaire items prior to data collection. Minor wording adjustments were made based on participant feedback to improve item clarity.

### 2.4 Procedure

Data collection was conducted in person through the administration of a paper-based questionnaire. Participants were recruited consecutively from four first-level public healthcare centers (“subcentros de salud”) belonging to the Ecuadorian Ministry of Public Health while attending routine pediatric or obstetric consultations during the study period.

Initial contact with potential participants was established in the waiting areas of the healthcare facilities prior to their scheduled medical consultations. At that time, trained members of the research team explained the objectives of the study, the voluntary nature of participation, and the confidentiality and anonymity of the collected information.

Mothers who agreed to participate signed written informed consent before receiving the questionnaire. The instrument was completed independently by the participants in a self-administered paper format. During questionnaire completion, members of the research team remained available to clarify questions related exclusively to the wording or comprehension of the items, without intervening in or influencing participants' responses, to minimize interviewer and information bias.

Questionnaire administration took place in an environment that ensured adequate privacy and confidentiality. The average completion time ranged between 11 and 15 min and did not interfere with routine medical care or scheduled consultations.

Completed questionnaires were reviewed immediately after administration to identify missing or incomplete responses. All collected data were anonymized and coded prior to statistical analysis. Questionnaires with substantial missing information were excluded from the final database prior to analysis.

### 2.5 Data analysis

Given the descriptive nature of the study and its presentation as a Brief Research Report, data were analyzed using descriptive statistics only. Categorical variables were summarized as absolute frequencies and percentages, while age was described using measures of central tendency and dispersion, according to its distribution.

To provide a more precise estimation of the observed proportions, 95% confidence intervals (95% CI) were calculated. The participation rate was 58.8% of the eligible population. Data analysis was performed using SPSS statistical software version 26.0 (IBM Corp., Armonk, NY, USA).

No inferential analyses or multivariable models were performed, as the objective of the study was to describe early breastfeeding practices within the first hour of life and perinatal conditions, without assessing associations or causal relationships. This analytical approach was considered appropriate given the descriptive nature of the study and its purpose as preliminary research on breastfeeding practices and early-life nutrition.

## 3 Results

### 3.1 Sociodemographic characteristics of participants

A total of 343 mothers participated in the study. The mean age was 29.22 years (SD = 6.25), and the median age was 29 years.

Most participants lived in urban areas (88.3%) and self-identified as mestizo (99.1%). Single women constituted the largest marital-status group (45.2%), followed by married women (32.4%). Secondary education was the most frequently reported educational level (42.0%), whereas 35.0% had completed undergraduate studies. Additional sociodemographic characteristics are shown in Table 1.

**Table 1:** Sociodemographic characteristics of the study participants.

Variable	Dimension	*N*	%	CI 95%	
				Lower	Upper
Age	M = 29.22 ± 6.25 Md = 29				
Marital status	Single	155	45.2	39.9	50.4
	Married	111	32.4	27.5	37.4
	Cohabiting (consensual union)	63	18.4	14.5	22.7
	Divorced	14	4.1	2.3	6.5
Ethnicity	Mestizo	340	99.1	97.6	99.7
	White	2	0.6	0.1	1.8
	Indigenous	1	0.3	0.03	1.3
Level of education	Incomplete primary education	19	5.5	3.4	8.3
	Primary education	40	11.7	8.5	15.3
	Secondary education	144	42	36.8	47.2
	Undergraduate level	120	35	30.1	40.1
	Postgraduate level	20	5.8	3.7	8.6
Place of residence	Urban	303	88.3	84.6	91.4
	Rural	40	11.7	8.5	15.3
Occupation prior to pregnancy	Student	76	22.2	18.0	26.8
	Employed	68	19.8	15.9	24.3
	Unemployed	80	23.3	19.1	28.0
	Public sector employee	24	7.0	4.7	10.1
	Self-employed	83	24.2	19.9	28.9
	Other (homemaker)	12	3.5	1.9	5.8

Data are presented as absolute frequencies (*n*), percentages (%), and corresponding 95% confidence intervals (95% CI). Percentages and confidence intervals were calculated based on the total study sample (*N* = 343). The table describes the sociodemographic characteristics of the study participants. CI, confidence interval; Md, median; SD, standard deviation.

### 3.2 Early breastfeeding practices during the first hour of life

Early initiation of breastfeeding, defined as initiation within the first hour of life, was reported in 56 % of participants. Conversely, 44.0% of mothers reported initiating breastfeeding after the first hour postpartum.

Regarding the duration of the first breastfeeding session, the most frequently reported duration was between 5 and 10 min (40.5%), followed by mothers who did not remember the duration (26.2%). A smaller proportion reported breastfeeding durations longer than 10 min or shorter than 5 min during the initial feeding session.

Immediate skin-to-skin contact was reported by 77.8% of mothers, while smaller proportions reported contact after 30 min or after 1 h.

Most participants (66.5%) reported no difficulties initiating breastfeeding during the first hour postpartum, whereas 33.5% reported experiencing some degree of difficulty during this period. Among mothers who reported difficulties, the most frequently mentioned challenges: pain when breastfeeding, anatomical alteration (inverted nipple), poor posture, weak suction. Additional breastfeeding practices and experiences during the first hour after birth are presented in Table 2.

**Table 2:** Early breastfeeding practices within the first hour of life.

Conditions	Category	*N*	%	IC 95%	
				Lower	Upper
Initiation of breastfeeding	Within the first hour	192	56	50.6	61.1
	After the first hour	151	44	38.8	49.3
Duration of the first breastfeeding session	Less than 5 min	61	17.8	13.7	21.8
	Between 5 and 10 min	139	40.5	35.3	45.6
	More than 10 min	53	15.5	11.6	19.3
	Does not remember	90	26.2	21.5	30.8
Skin-to-skin contact	Immediate skin-to-skin contact	267	77.8	73.2	81.9
	After 30 min	8	2.3	1.1	4.3
	After 1 h	68	19.8	15.8	24.2
Difficulty breastfeeding during the first hour after birth	Yes	115	33.5	28.6	38.6
	No	228	66.5	61.3	71.3
Main causes of difficulty breastfeeding during the first hour after birth	Pain when breastfeeding	49	14.3	10.9	18.3
	Anatomical alteration (inverted nipple)	32	9.3	6.6	12.7
	Poor posture	22	6.4	3.8	8.9
	Weak suction	12	3.5	1.9	5.8

Data are presented as absolute frequencies (*n*), percentages (%), and corresponding 95% confidence intervals (95% CI). Percentages and confidence intervals were calculated based on the total study sample (*N* = 343). The table describes breastfeeding initiation, duration of the first breastfeeding session, skin-to-skin contact, and breastfeeding difficulties during the first hour postpartum. CI, confidence interval.

### 3.3 Delivery characteristics and perinatal conditions in the study population

Vaginal delivery accounted for 51.9% of births, while cesarean delivery represented 48.1%.

Maternal complications were reported in 21.0% of participants. Among affected mothers, preterm labor was the most frequently reported complication, followed by postpartum hemorrhage and perineal tear. Most participants, however, did not report maternal complications during delivery or the immediate postpartum period. Neonatal complications were reported in 12.2% of newborns. Among affected newborns, prematurity was the most frequently reported complication (33.0%), followed by meconium-stained amniotic fluid (21.0%) and respiratory complications (19.0%). Additional delivery characteristics and perinatal conditions are presented in Table 3.

**Table 3:** Delivery conditions and perinatal factors.

Conditions	Category	*N*	%	IC 95%	
				Lower	Upper
Type of delivery	Vaginal delivery	178	51.9	46.6	57.1
	Cesarean delivery	165	48.1	42.9	53.4
Maternal complications	Yes	72	21	16.9	25.5
	No	271	79	74.5	83.1
Main maternal complications^*^	Preterm labor	15	20.8	11.44	30.2
	Perineal tear	7	10	3.1	16.9
	Postpartum hemorrhage	8	11	3.77	18.22
	Retained placenta	2	3	0.94	6.94
Neonatal complications^*^	Yes	42	12.2	9.1	16
	No	301	87.8	84	90.9
	Meconium-stained amniotic fluid	9	21	8.6	33.3
	Nuchal cord	6	14	3.5	24.4
	Need for resuscitation	5	12	2.1	21.8
	Prematurity	14	33	18.7	47.2
	Respiratory complications	8	19	7.1	30.8

Data are presented as frequencies (*n*), percentages (%), and 95% confidence intervals (95% CI). Percentages for type of delivery and the presence of maternal and neonatal complications were calculated using the total study population (*N* = 343) as the denominator. Percentages for specific maternal complications were calculated among mothers reporting maternal complications (*n* = 72), whereas percentages for specific neonatal complications were calculated among newborns with neonatal complications (*n* = 42). Categories marked with an asterisk (^*^) represent the most frequently reported complications. CI, confidence interval.

### 3.4 Descriptive distribution of breastfeeding initiation timing across selected perinatal conditions

Among mothers who had a vaginal delivery, 82.0% reported breastfeeding initiation within the first hour of life, whereas 18.0% reported delayed initiation. In contrast, among mothers who underwent cesarean delivery, 28.0% reported early initiation and 72.0% reported delayed initiation.

Early initiation was reported by 58.8% of mothers without maternal complications and by 41.7% of those who reported maternal complications, whereas delayed initiation was reported by 40.2% and 58.3%, respectively.

Regarding neonatal conditions, early initiation was reported by 59.8% of mothers whose newborns did not experience neonatal complications and by 28.6% of mothers whose newborns experienced neonatal complications. Delayed initiation was reported by 40.2% and 71.4%, respectively.

Among mothers who reported immediate skin-to-skin contact after birth, 67% reported breastfeeding initiation within the first hour of life, whereas 33% reported delayed initiation. Among mothers whose skin-to-skin contact occurred after 30 min, 62.5% reported early initiation and 37.5% reported delayed initiation. Among mothers whose skin-to-skin contact occurred after 1 h, 11.8% reported early initiation and 88.2% reported delayed initiation Table 4.

**Table 4:** Maternal latency stratified by major perinatal factors.

Conditions	Category	Early BF initiation			Delayed BF initiation		
		*N*	%	IC 95%	N	%	95% CI
Type of delivery	Vaginal delivery	146	82	75.9–87.1	32	18	12.9–24.1
	Cesarean delivery	46	28	21.5–35.1	119	72	64.9–78.5
Maternal complications	Yes	30	41.7	30.8–53.2	42	58.3	46.8–69.2
	No	162	59.8	53.9–65.5	109	40.2	34.5–46.1
Neonatal complications^*^	Yes	12	28.6	16.7–43.3	30	71.4	56.7–83.3
	No	180	59.8	54.2–65.2	121	40.2	34.8–45.8
Skin-to-skin contact^*^	Immediate skin-to-skin contact	179	67	61.2–72.3	91	33	27.5–38.8
	After 30 min	5	6.5	29.5–88.1	3	37.5	11.9–70.5
	After 1 h	8	11.8	5.7–21.0	60	88.2	79.0–94.3

Data are presented as frequencies (*n*), percentages (%), and 95% confidence intervals (95% CI). Percentages were calculated within each category of the corresponding perinatal condition. Early breastfeeding initiation (EIBF) was defined as breastfeeding initiated within the first hour after birth, whereas delayed breastfeeding initiation was defined as breastfeeding initiated after the first hour. Categories marked with an asterisk (^*^) represent selected perinatal conditions reported by participants. CI, confidence interval.

## 4 Discussion

The present study provides descriptive evidence on breastfeeding practices during the first hour after birth among mothers of children younger than 2 years who were recruited from four primary healthcare centers in southern Ecuador. Participants retrospectively reported their breastfeeding experiences and perinatal circumstances surrounding childbirth. More than half of the mothers reported initiating breastfeeding within the first hour of life, whereas a substantial proportion reported delayed initiation. As early initiation of breastfeeding remains one of the most widely monitored indicators of newborn care recommended by international organizations, the prevalence observed in this cohort provides relevant information regarding breastfeeding practices within the study population (2, 3, 6, 16).

The prevalence of early breastfeeding initiation observed in the present study falls within the broad range reported internationally. Studies conducted in Latin America, Asia, and sub-Saharan Africa have documented considerable variability in rates of breastfeeding initiation during the first hour after birth, with prevalences ranging from less than 40% to more than 70% (7–11, 17–29). Similar heterogeneity has been described in Brazil, Colombia, Mexico, Bangladesh, India, and several African countries (8–11, 18–29). Evidence from high-income and other international settings further extends this perspective. In Europe and Central Asia, recent regional estimates indicate that approximately 53% of newborns are breastfed within the first hour after birth (30). In Australia, a recent study among women following cesarean birth reported that 70.2% initiated breastfeeding within the first hour after birth (31).

In the United States, recent evidence indicates that early breastfeeding-supportive practices are frequently implemented during the immediate postpartum period. The combined practice of skin-to-skin contact and breastfeeding within the first hour after birth was reported in approximately 70–82% of mothers across different population groups, suggesting that early breastfeeding is commonly supported in U.S. maternity-care settings, although variability across groups remains (32). In Canada, recent national surveillance indicates high breastfeeding initiation, reaching approximately 91%. However, this indicator reflects breastfeeding initiation regardless of timing and does not specifically distinguish initiation within the first hour after birth, limiting direct comparison with the WHO-defined EIBF indicator (33).

Taken together, these findings place the prevalence observed in southern Ecuador within a broader international context and highlight the considerable variability in early breastfeeding initiation across populations and healthcare settings. The present study therefore contributes context-specific evidence from southern Ecuador to the global understanding of breastfeeding practices during the first hour after birth.

An important aspect of the present findings is that breastfeeding initiation occurred within a perinatal context characterized by a high frequency of immediate skin-to-skin contact, a substantial proportion of cesarean deliveries, maternal reports of breastfeeding difficulties, and the presence of maternal and neonatal complications. Considered together, these findings illustrate the complexity of the circumstances surrounding breastfeeding initiation during the immediate postpartum period and highlight the diversity of breastfeeding experiences reported by participating mothers.

Although no inferential analyses were performed, substantial differences in the descriptive distribution of breastfeeding initiation were observed across selected perinatal conditions. Early initiation of breastfeeding was reported by 82.0% of mothers who experienced vaginal delivery compared with 28.0% of those who underwent cesarean delivery. Likewise, early initiation was reported by 67.0% of mothers who experienced immediate skin-to-skin contact, whereas only 11.8% of mothers whose skin-to-skin contact occurred after 1 h reported breastfeeding initiation within the first hour. Similar differences were observed according to the presence of neonatal complications. These findings should not be interpreted as evidence of statistical associations. Nevertheless, they highlight substantial variability in the descriptive distribution of breastfeeding initiation across different perinatal conditions and provide a broader understanding of the context in which breastfeeding was established among participating mothers.

One of the most favorable findings was the high frequency of immediate skin-to-skin contact reported by participants. This practice is widely promoted as part of essential newborn care and breastfeeding support strategies (13, 14, 16, 34–37). However, despite the high frequency of immediate skin-to-skin contact, a substantial proportion of mothers reported delayed breastfeeding initiation. Although the descriptive design of the study does not allow evaluation of the factors underlying this pattern, the coexistence of these findings suggests that breastfeeding initiation during the first hours after birth occurs within a context influenced by multiple clinical and healthcare-related circumstances. These results reinforce the importance of continuing to monitor breastfeeding practices during the immediate postpartum period.

Approximately one-third of participating mothers reported difficulties initiating breastfeeding during the first hour after birth. The most frequently reported difficulties included breastfeeding-related pain, inverted nipples, poor positioning, and weak infant sucking. Although these experiences were not evaluated in relation to subsequent breastfeeding outcomes, their frequency indicates that challenges during the immediate postpartum period remain relatively common. Identifying these experiences contributes to a broader understanding of maternal perceptions and breastfeeding practices during a period considered critical for the establishment of infant feeding (16, 22, 38, 39).

The high frequency of cesarean deliveries observed in this cohort also deserves consideration. Nearly half of all births occurred by cesarean section, a proportion comparable to that reported in several maternal healthcare settings worldwide (26–29, 40–43).

In addition to its obstetric relevance, documenting the frequency of cesarean births contributes to a more comprehensive characterization of the perinatal circumstances surrounding breastfeeding initiation in the study population. Given the descriptive nature of the study, the clinical indications for cesarean delivery and other healthcare-related factors were not evaluated; therefore, no conclusions can be drawn regarding their influence on breastfeeding initiation.

Maternal and neonatal complications were also reported by a proportion of participants and formed part of the clinical context surrounding breastfeeding initiation. Maternal complications were reported by approximately one-fifth of mothers, whereas neonatal complications were reported in slightly more than one in ten newborns. Prematurity was the most frequently reported neonatal complication. The identification of these conditions contributes to the characterization of the maternal and neonatal care environment surrounding breastfeeding initiation and provides additional context for understanding the diversity of breastfeeding experiences reported by participating mothers (1, 16, 44).

Overall, the findings describe a population in which early breastfeeding initiation was reported by more than half of participating mothers, within a perinatal context characterized by frequent skin-to-skin contact, a substantial proportion of cesarean births, and the presence of diverse maternal and neonatal conditions. From a maternal and child health perspective, continued monitoring of breastfeeding initiation practices remains relevant for understanding how breastfeeding is established in different populations and healthcare contexts. The present study contributes descriptive evidence regarding breastfeeding practices among mothers of children younger than 2 years recruited through primary healthcare centers in southern Ecuador and provides a foundation for future analytical research aimed at better understanding the factors and healthcare circumstances associated with breastfeeding initiation during the immediate postpartum period.

## 5 Implications for clinical practice and public health

The present study provides a comprehensive descriptive characterization of breastfeeding practices during the first hour of life and the perinatal circumstances reported by mothers recruited through public primary healthcare centers in southern Ecuador. As recommended by the World Health Organization and UNICEF, early initiation of breastfeeding is widely monitored as an important component of maternal and newborn care (2, 6). In this context, the descriptive stratification performed in the present study identified substantial differences in the distribution of early breastfeeding initiation across selected perinatal conditions, including mode of delivery, timing of skin-to-skin contact, and the presence of maternal and neonatal complications. Although the study was not designed to evaluate statistical associations or determinants of breastfeeding initiation, these findings contribute to a more comprehensive characterization of the perinatal circumstances in which breastfeeding initiation occurred within the study population. Together with the existing evidence, the present results provide locally generated descriptive information that may support the continued monitoring of breastfeeding practices and inform future analytical studies exploring breastfeeding initiation in different healthcare settings.

## 6 Limitations

This study has several limitations that should be considered when interpreting the findings.

First, the descriptive cross-sectional design precludes causal and temporal inferences. Consequently, the findings should be interpreted as descriptive estimates of breastfeeding practices and perinatal characteristics observed in the study population.

Second, the study was conducted in four public primary healthcare centers in the city of Loja, southern Ecuador. Although the study sought to recruit all eligible mothers registered in these centers, the findings reflect the characteristics of this specific population and should not be considered representative of the Ecuadorian population.

Third, data were obtained through maternal self-report using a retrospective questionnaire. Therefore, some variables may be subject to recall bias, particularly those related to events occurring during the immediate postpartum period. Although participants were allowed to consult their Maternal and Child Health Record Card when clarification of perinatal information was required, information was not systematically verified against clinical records. Furthermore, the time elapsed since childbirth was not recorded, preventing assessment of possible differences in recall accuracy according to the child's age at the time of the interview.

Fourth, although the questionnaire underwent content validation through expert review and a pilot comprehension assessment involving 50 mothers, formal reliability testing was not performed. This was because the instrument was designed to collect heterogeneous descriptive information across multiple domains, including sociodemographic characteristics, obstetric history, perinatal conditions, and breastfeeding practices, rather than to measure a single latent construct for which internal consistency would be expected.

Finally, the study did not evaluate healthcare facility-level factors that may influence breastfeeding initiation, such as institutional protocols, staffing practices, or breastfeeding support strategies. In addition, no inferential analyses were performed; therefore, the findings should not be interpreted as evidence of statistical associations.

## 7 Conclusions

In conclusion, slightly more than half of the mothers in this southern Ecuadorian cohort reported initiating breastfeeding within the first hour after birth, while a substantial proportion reported delayed initiation. These findings occurred within a perinatal context characterized by frequent immediate skin-to-skin contact, a high proportion of cesarean births, and reported maternal and neonatal conditions. When placed within the broader international evidence, the prevalence observed in this population should be interpreted in the context of the considerable variation in early breastfeeding initiation reported across populations and healthcare settings. This study provides context-specific descriptive evidence from southern Ecuador and underscores the value of continued monitoring of breastfeeding practices during the immediate postpartum period. Future analytical studies are needed to examine maternal, neonatal, and healthcare-related factors associated with early breastfeeding initiation in this and other healthcare settings.

## Data Availability

The original contributions presented in the study are included in the article/Supplementary material, further inquiries can be directed to the corresponding author.
